# Co-Analysis of Transcriptome and Metabolome Reveals Anthocyanin Accumulation in the Female Flower Tissues of Fig Cultivar ‘Silu Hongyu’

**DOI:** 10.3390/genes17060694

**Published:** 2026-06-14

**Authors:** Ying Zhang, Yuqin Jiang, Shuanti Qian, Siyu Jing, Zijin Liu, Zhihao Zhao

**Affiliations:** 1 College of Ecological and Environmental Engineering, Shaanxi A&F Technology University, Yangling 712100, China; qianshuanti@yeah.net (S.Q.); siyujing11@163.com (S.J.); 2 College of Agronomy, Northwest A&F University, Yangling 712100, China; jyq32322021@nwafu.edu.cn (Y.J.); liuzijin@nwafu.edu.cn (Z.L.); 3 Chinese Academy of Tropical Agricultural Sciences, Haikou 571101, China

**Keywords:** Fig, anthocyanin, metabolome, transcriptome, female flower tissues

## Abstract

Background/Objectives: Fig (*Ficus carica* L.) is considered a valuable fruit owing to its rich health-promoting ingredients, including anthocyanins. However, little information is available on the regulatory networks that reveal anthocyanin biosynthesis in figs, especially the new fig cultivar “Silu Hongyu” (HY). Methods: In this study, multi-omics analysis was performed to dissect the regulatory networks responsible for anthocyanin accumulation in the female flower tissues of HY. Results: we found that the anthocyanin content in the female flower tissues of HY is higher than that of “Chinese Ziguo” (ZG). Metabolomic profiling identified 350 differentially accumulated metabolites (DAMs), among which 108 were flavonoids. The contents of multiple metabolites responsible for anthocyanin accumulation, such as naringenin chalcones, cyanidin 3-glucoside, and pelargonidin 3,5-diglucoside, were significantly increased in the HY female flower tissues. Transcriptomic analysis revealed that 3696 differentially expressed genes (DEGs) were screened from the female flower tissues of ZG and HY, with 1730 upregulated DEGs and 1966 downregulated DEGs in HY compared to ZG. The key structural genes involved in anthocyanin biosynthesis, including *FcPAL*, *Fc4CL*, *FcCHS*, *FcF3′H*, and *FcBZ1*, were significantly upregulated in the female flower tissues of HY compared with ZG. KEGG analysis also demonstrated that five flavonoid biosynthesis pathways were co-enriched by DAMs and DEGs. Conclusion: These findings provide a multi-omics framework that governs anthocyanin biosynthesis in the female flower tissues of HY, which will facilitate the genetic breeding and improvement of high-anthocyanin fig cultivars.

## 1. Introduction

Fig (*Ficus carica* L.), one of the earliest domesticated fruit tree species globally, is taxonomically classified under the Moraceae family and commercially cultivated as a high-value cash crop across the Mediterranean basin, the United States, China, Japan, and multiple regions of the Southern Hemisphere [1]. Having been introduced to China via the ancient Silk Road several millennia ago [2], this fruit has long been integrated into local agricultural systems and dietary cultures. Beyond its widespread consumption attributed to its palatable organoleptic properties and abundant mineral nutrients, fig is increasingly recognized for its diverse bioactive constituents, which confer significant antioxidant, antibacterial, and anticancer activities, as well as antihypertensive and hypolipidemic effects [2,3,4]. Botanically, the edible structure of figs is derived from female flowers within an inflorescence enclosed by a fleshy receptacle, thereby defining it as a false fruit designated as a ‘syconium’ [5]. The syconium exhibits substantial versatility in consumption, being readily consumed fresh, dried, or processed into a variety of value-added products. Notably, distinct from major cultivars of common fruits such as apple (*Malus domestica*), orange (*Citrus sinensis*), peach (*Prunus persica*), and pear (*Pyrus communis*), which typically produce anthocyanin-free flesh, fig flesh is able to accumulate high levels of anthocyanins, a class of bioactive flavonoids with considerable nutritional and functional significance [4].

Anthocyanins are a diverse group of water-soluble polyphenolic pigments responsible for the red, blue, purple, and pink coloration of plant flowers, seeds, fruits, and vegetative tissues [6]. Besides enhancing plant visual attractiveness, they play vital physiological roles in stress resistance and pollinator attraction [7,8]. Accordingly, these pigments possess considerable application potential in numerous fields. To date, over 600 anthocyanin variants have been identified in plant species [9]. Anthocyanins are biosynthesized via the phenylpropanoid pathway, and their biosynthesis has been well documented in vascular plants [9,10]. Starting with phenylalanine as the initial precursor, a series of colorless intermediates are sequentially generated through the catalytic actions of phenylalanine ammonia-lyase (PAL), cinnamic acid 4-hydroxylase (C4H), 4-coumarate:coenzyme A ligase (4CL), chalcone synthase (CHS), chalcone isomerase (CHI), flavanone 3-hydroxylase (F3H), flavanone 3′-hydroxylase (F3′H), flavonoid 3′5′-hydroxylase, and dihydroflavonol 4-reductase (DFR). Subsequently, unstable colored anthocyanidins are produced from these colorless precursors by anthocyanidin synthase (ANS). Finally, these unstable anthocyanidins are modified into stable blue-violet, brick-red, or magenta anthocyanins by UDP-glucose: flavonoid 3-O-glucosyltransferase (UFGT) [6,9,10]. Notably, brightly pigmented fruits and vegetables typically exhibit high transcriptional levels of key downstream structural genes in the anthocyanin biosynthetic pathway, including those encoding DFR, ANS, and UFGT [4,11,12,13]. Additionally, anthocyanin biosynthesis is also subject to sophisticated transcriptional control by transcription factors (TFs). Accumulating evidence indicates that TFs including MYB, basic helix–loop–helix (bHLH), and WD40 family members can activate the expression of structural genes involved in the anthocyanin biosynthetic pathway [10,14,15,16]. Moreover, several MYB TFs with repressive functions on these structural genes have also been characterized in plants [17,18,19]. Beyond these well-studied families, an expanding array of novel TFs belonging to other gene families have been continuously identified and documented in fruit species [20,21,22]. However, the structural genes and TFs governing anthocyanin biosynthesis in fig female flower tissues remain largely uncharacterized.

Recent advances in high-throughput omics technologies represented by transcriptomics and metabolomics have offered robust approaches for systematically identifying key regulatory genes and metabolites involved in plant tissue pigmentation [12,23,24,25]. Here, we employed an integrated transcriptomic and metabolomic approach to elucidate the molecular mechanisms driving metabolite differences in female flower tissues between two representative fig cultivars with distinct anthocyanin accumulation profiles. Our multi-omics results identified a suite of functional elements governing color formation in these tissues, yielding novel insights into the regulatory basis of their pigmentation. This study thus establishes a valuable theoretical foundation for the genetic improvement of fig fruit quality.

## 2. Results

### 2.1. Anthocyanin Contents in Female Flower Tissues of Figs

**Figure 1:**
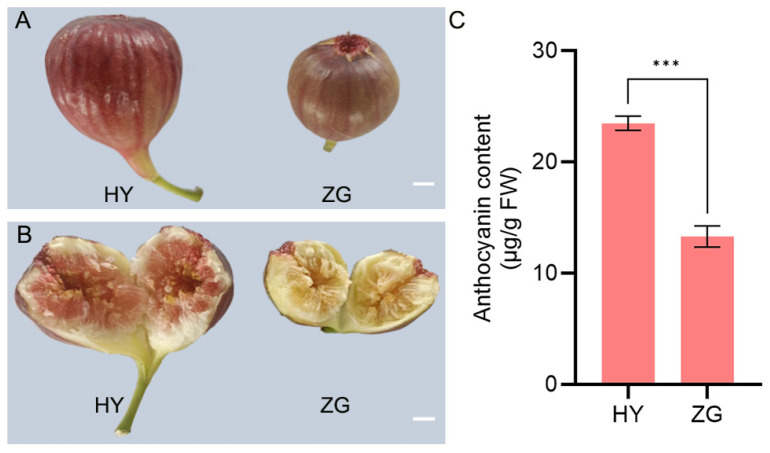
The phenotypes (**A**,**B**) and anthocyanin contents (**C**) of the female tissues in HY and ZG. HY, ‘Silu Hongyu’; ZG, ‘Chinese Ziguo’; FW, fresh weight; Bars = 1 cm. Values are means ± SD (*n* = 3), and statistically significant differences between the HY and ZG (Student’s *t*-test, *** *p* < 0.001) are indicated by asterisks.

The ‘Silu Hongyu’ (HY) is a fig cultivar introduced and domesticated by our team in Yangling, China. ‘Chinese Ziguo’ (ZG) possesses excellent agronomic traits such as high yield, cold tolerance, and ease of cultivation. To compare the phenotypic and biochemical characteristics between the two cultivars, we analyzed their fruit morphology and anthocyanin accumulation at the ripening stage (80 days after fruit setting). As shown in Figure 1A, obvious differences in fruit size were observed between the two cultivars, with HY exhibiting significantly larger longitudinal and transverse diameters than ZG (Figure 1A). Upon halving the fruits, the internal female flower tissues of HY exhibited a pink–garnet hue, whereas those of ZG appeared pale yellow (Figure 1B). Quantification of anthocyanin content further showed that the female flower tissues of HY accumulated higher anthocyanin levels than those of ZG (Figure 1C). These results demonstrate that HY is a high-anthocyanin fig cultivar.

### 2.2. Metabolic Profiling and Multivariate Statistical Analysis

**Figure 2:**
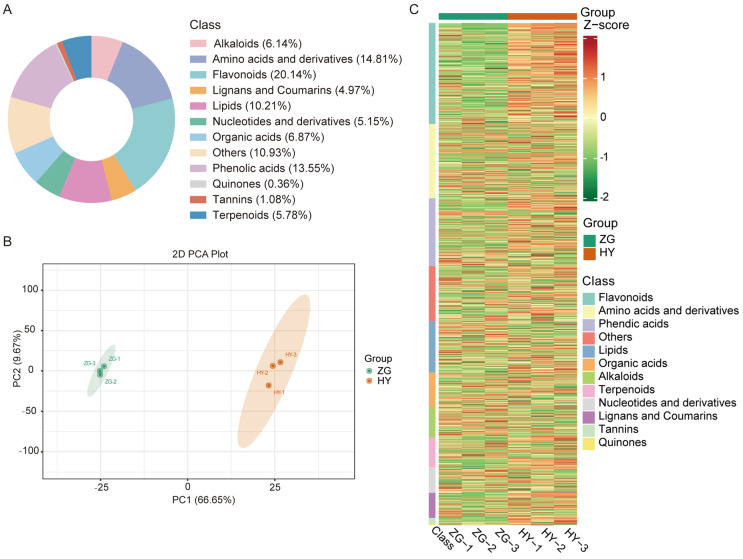
Preliminary analysis of metabolomics data of the female flower tissues from ZG and HY. (**A**) Circular plot depicting metabolite categories. (**B**) Principal component analysis (PCA) plot of metabolomic profiles. (**C**) Hierarchical clustering analysis of metabolites. The color indicates the accumulation level of each metabolite, from low (green) to high (red). ZG and HY represent two fig varieties ‘Chinese Ziguo’ and ‘Silu Hongyu’.

To further investigate the mechanism underlying the differences in anthocyanin content between the two fig varieties, we profiled the metabolome of female flower tissues from ZG and HY using a widely targeted metabolomics approach. A total of 1107 metabolites were determined and grouped into 12 classes, including of alkaloids (68, 6.14%), amino acids and derivatives (164, 14.81%), flavonoids (223, 20.14%), lignans and coumarins (55, 4.97%), lipids (113, 10.21%), nucleotides and derivatives (57, 5.15%), organic acids (76, 6.87%), phenolic acids (150, 13.55%), quinones (4, 0.36%), tannins (12, 1.08%), terpenoids (64, 5.78%), and others (121, 10.93%) (Figure 2A, Table S1). Principal component analysis (PCA) with an unsupervised model was performed to analyze the identified metabolites and evaluate overall differences among samples. We observed that samples within each group were tightly clustered, indicating good repeatability (Figure 2B). In the PCA score plot, two principal components explained 76.32% of the total variance, with the first principal component (PC1) and the second PC2 contributing 66.65% and 9.67% of the variance, respectively (Figure 2B). The six samples from the two fig varieties were distinctly clustered into two groups, and each group exhibited high cohesion (Figure 2B). Hierarchical cluster analysis (HCA) also showed that all biological replicates of ZG and HY were grouped, indicating a high reliability of the generated metabolome data (Figure 2C). Meanwhile, metabolites were classified into 12 major clusters based on their relative contents (Figure 2C). Among these clusters, flavonoid metabolites accumulated to significantly higher levels in HY than in ZG (Figure 2C).

Taken together, these results confirm significant differences in metabolite profiles between HY and ZG.

### 2.3. Identification and KEGG Enrichment Analysis of Differentially Accumulated Metabolites (DAMs)

**Figure 3:**
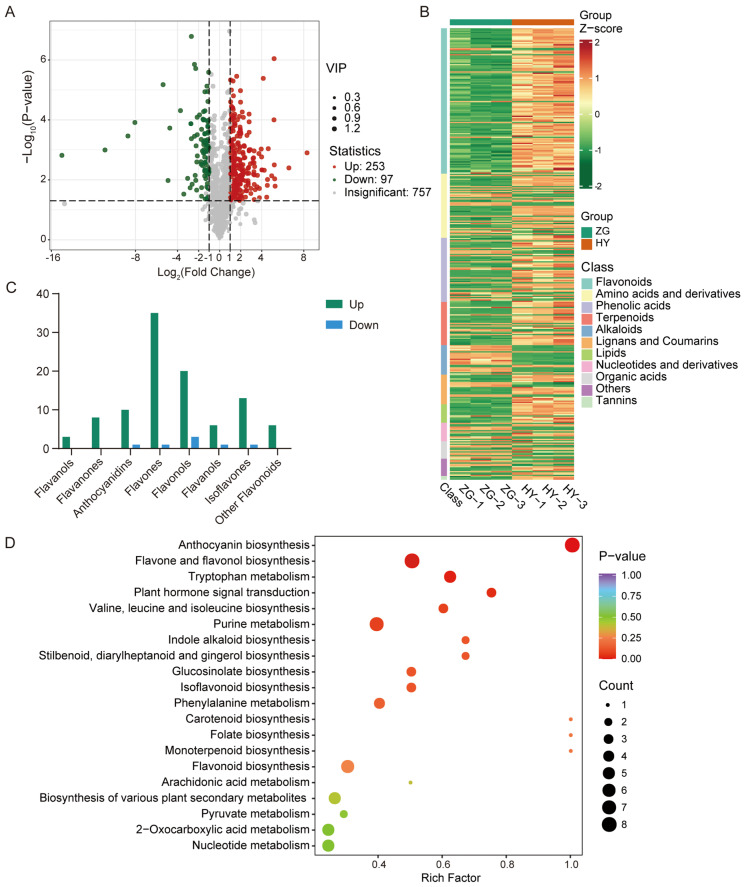
Identification and analysis of the differentially accumulated metabolites (DAMs) between two fig varieties ‘Chinese Ziguo’ (ZG) and ‘Silu Hongyu’ (HY). (**A**) Volcano plot depicting differential metabolite accumulation levels. VIP, variable importance in the projection. (**B**) Heatmap clustering of DAMs. The color indicates the level of accumulation of each metabolite, from low (green) to high (red). (**C**) The numbers of upregulated and downregulated flavonoids. (**D**) KEGG enrichment analysis of the DAMs between ZG vs HY.

The DAMs between ZG and HY were identified based on the variable importance in absolute log_2_ fold change (|log_2_FC|) ≥ 1, variable importance in projection (VIP > 1) and *p* < 0.05. There were 350 DAMs between ZG vs HY, including of 253 upregulated and 97 downregulated DAMs (Figure 3A, Table S2). Further HCA analysis of the DAMs showed that DAMs were categorized into 11 subgroups, with flavonoids being the first major subgroup and showing significantly higher levels in the female flower tissues of HY than in those of ZG (Figure 3B). A total of 108 differentially accumulated flavonoids were screened, including three chalcones, eight flavanones, 11 anthocyanins, 36 flavones, 23 flavonols, seven flavanols, 14 isoflavones and six other flavonoids (Figure 3C, Table S3). A Kyoto Encyclopedia of Genes and Genomes (KEGG) enrichment analysis also showed that anthocyanin biosynthesis (ko00942), flavone and flavonol biosynthesis (ko00944), isoflavonoid biosynthesis (ko00943), and flavonoid biosynthesis (ko00941) were significantly enriched (*p* < 0.05) (Figure 3D, Table S4). These results imply that the DAMs from the flavonoid biosynthesis pathway are responsible for the color difference of female flower tissues between HY and ZG.

### 2.4. Overview of the Transcriptome Sequencing of ZG and HY

**Figure 4:**
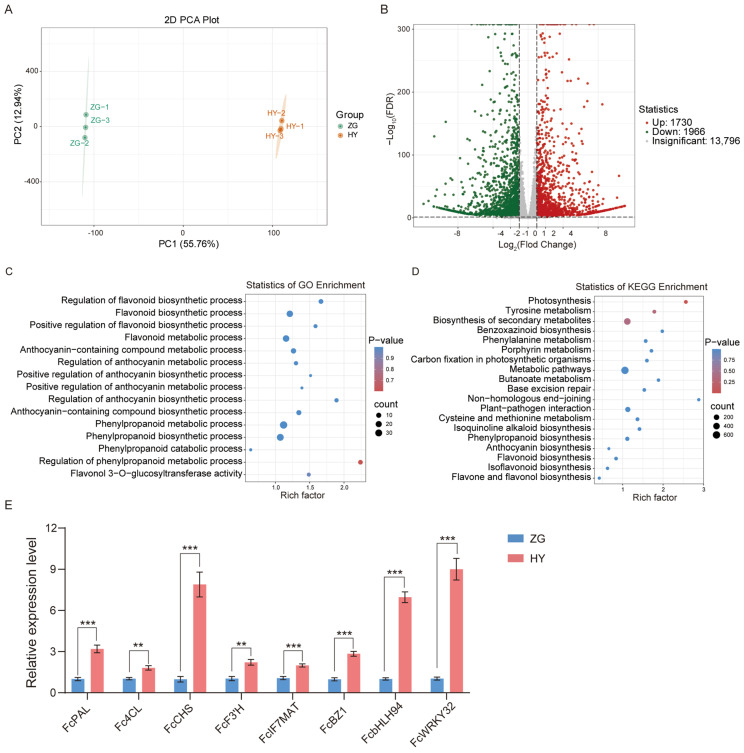
Differentially expressed genes (DEGs) in the female flower tissues between ‘Chinese Ziguo’ (ZG) and ‘Silu Hongyu’ (HY). (**A**) Principal component analysis (PCA) plot of transcriptome profiles. (**B**) Volcano plot depicting DEGs between ZG and HY. (**C**,**D**) Bubble plot illustrating the enrichment of gene ontology (GO) (**C**) and KEGG pathways (**D**) associated with flavonoid biosynthesis and metabolism. Bubble size represents the number of genes enriched in this pathway, while color intensity indicates the statistical significance of enrichment. (**E**) RT-qPCR analysis of 8 DEGs related to flavonoid biosynthesis in female flower tissues of ZG and HY. Total RNA was isolated from female flower tissues at about 75 days after fruit setting. Results were normalized against the expression of *Actin* as an internal control. Asterisks indicate significant differences in gene expression levels between HY and ZG (two-tailed paired Student’s *t*-test, ** *p* < 0.01; *** *p* < 0.001). Values are means ± SD (*n* = 3).

To explore the molecular mechanisms underlying the enhanced accumulation of anthocyanins in the female flower tissues of HY, we constructed six cDNA libraries for transcriptomic analysis. The total number of raw reads generated in each library ranged from 47,169,226 to 57,514,186. After filtering the raw data, the total number of clean reads per library was from 46,260,756 to 56,482,152 with a Q30 percentage of over 96.53% (0.02% error rate) and a GC percentage of average 46.72% (Table S5), indicating that the reads were of high quality and could be used for differential gene expression analysis. Of the total clean reads, 74.44%~92.22% matched with the *F. carica* reference genome (Table S5). PCA showed that all the biological replicates clustered together, and an obvious separation was observed between ZG and HY samples (Figure 4A), implying the high reliability of our sequencing data.

### 2.5. Gene Ontology (GO) and KEGG Pathway Analyses of Differentially Expressed Genes (DEGs)

With criteria |log_2_FC| ≥ 1 and false discovery rate (*FDR*) < 0.05, 3696 DEGs were screened from the female flower tissues of ZG and HY, with 1730 upregulated genes and 1966 downregulated genes in HY compared to ZG (Figure 4B, Table S6). A focused analysis of flavonoid-associated processes revealed that a total of 61 DEGs were enriched in these processes (Figure 4C, Table S7). Further KEGG analysis showed that there are 72 DEGs enriched in the phenylpropanoid biosynthesis (ko00940, 52), anthocyanin biosynthesis (ko00942, 1), flavonoid biosynthesis (ko00941, 18), isoflavonoid biosynthesis (Ko00943, 8), and flavone and flavonol biosynthesis (ko00944, 3) (Figure 4D, Table S8). We randomly selected eight DEGs involved in flavonoid biosynthesis-related pathways to detect their expression levels in ZG and HY using reverse transcription quantitative polymerase chain reaction (RT-qPCR). The results showed that these genes were upregulated in female flower tissues of HY compared with ZG, and their expression patterns were consistent with the RNA sequencing (RNA-seq) results (Figure 4E, Table S6), validating the reliability of the transcriptomic data.

Collectively, these results suggest that the differential expression of these genes may underlie the distinct anthocyanin accumulation patterns observed in the female flower tissues between the two fig cultivars.

### 2.6. Correlation Analysis Between Transcripts and Metabolites

**Figure 5:**
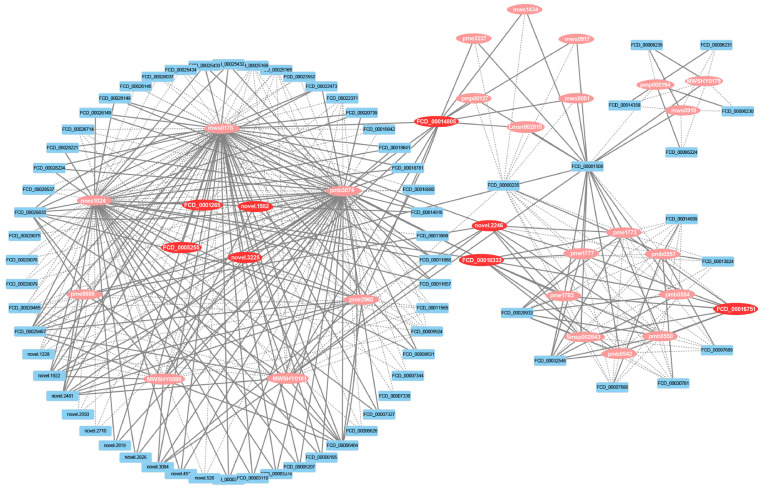
Connection networks of genes and metabolites involved in anthocyanin accumulation. Blue rectangles represent genes; pink ellipses represent different accumulations of flavonoid metabolites. Red diamonds represent key genes. Edges colored in ‘solid’ and ‘dashes’ represent positive and negative correlations, respectively.

To clarify the major metabolites and corresponding genes that cause differences in anthocyanin contents in female flower tissues between HY and ZG, we conducted a correlation analysis between gene expression profiles and metabolite contents. A rigorous multiple test correction (|coefficient| > 0.8, *FDR* < 0.05) was used to filter the genes significantly correlated with each metabolite. A total of 3647 genes were co-regulated with 92 flavonoid metabolites (Table S9). Mapping these highly correlated metabolites and genes to the KEGG database, we obtained 69 genes and 24 metabolites enriched in the phenylpropanoid biosynthesis (ko00940), flavonoid biosynthesis (ko00941), anthocyanin biosynthesis (ko00942), isoflavonoid biosynthesis (ko00943), flavone and flavonol biosynthesis (ko00944) pathways (Table S9). The DEGs and DAMs in female flower tissues of ZG and HY, such as chlorogenic acid (3-*O*-Caffeoylquinic acid, mws0178), p-Coumaraldehyde (mws1024), 5-*O*-p-Coumaroylquinic acid (pmb3074), were found to be related to phenylpropanoid biosynthesis. The 5-*O*-p-Coumaroylquinic acid (pmb3074), apigenin-8-C-Glucoside (vitexin, MWSHY0181), 5,7,3′,4′-Tetrahydroxyflavone (luteolin, pme0088), 2′,4,4′,6′-Tetrahydroxychalcone (pme2960), epigallocatechin (MWSHY0098), and chlorogenic acid (3-*O*-Caffeoylquinic acid, mws0178) were found to be related to flavonoid biosynthesis (ko00941). The pelargonidin-3-*O*-rutinoside (smsp002643), cyanidin-3,5-*O*-diglucoside (cyanin, pme1777), cyanidin-3-*O*-glucoside (kuromanin, pmb0550), cyanidin-3-*O*-(3″,6′’-*O*-dimalonyl) glucoside (pmb0557), cyanidin-3-*O*-(6″-*O*-malonyl) glucoside (pmb0542), pelargonidin-3,5-*O*-diglucoside (pme1793), cyanidin-3-*O*-rutinoside (keracyanin, pme1773), and pelargonidin-3-*O*-(6″-O-malonyl) glucoside (pmb0554) were found to belong to the differential anthocyanin metabolites in the ko00942 pathway. The prunetin (5,4′-Dihydroxy-7-methoxyisoflavone, mws0918), 6″-*O*-Malonylgenistin (pmp000194), and formononetin-7-*O*-glucoside (ononin, MWSHY0179) were related to isoflavonoid biosynthesis (ko00943). The 3,7-Di-*O*-methylquercetin (mws0917), acacetin (mws0051), apigenin-8-C-Glucoside (vitexin, MWSHY0181), Kaempferol-3-*O*-rutinoside (Nicotiflorin, Lmsn002815), luteolin (5,7,3′,4′-Tetrahydroxyflavone, pme0088), 5,7,4′-Trihydroxy-3′-Methoxyflavone (pmp001127), apigenin-6-C-glucoside (isovitexin, mws1434), and vitexin-2″-*O*-rhamnoside (pme3227) were found to be related to flavone and flavonol biosynthesis (ko00944) (Table S9). Correlation analysis showed that *FCD_00001265* and *FCD_00005255* are highly positively correlated with chlorogenic acid (3-*O*-Caffeoylquinic acid) (mws0178), p-Coumaraldehyde (mws1024), and 5-*O*-p-Coumaroylquinic acid (pmb3074). *FCD_00018333* (*UFOG5*), *novel.2246* (*FLS*), *FCD_00014805* (*F3*′*H*), and *FCD_00001500* (*MAT1*) exhibited simultaneous correlations with metabolites from two to three distinct pathways (Figure 5). This suggests that these enzymes are key in the flavonoid metabolic network.

### 2.7. A Joint Transcriptomic and Metabolic Analysis of Flavonoid Accumulation

**Figure 6:**
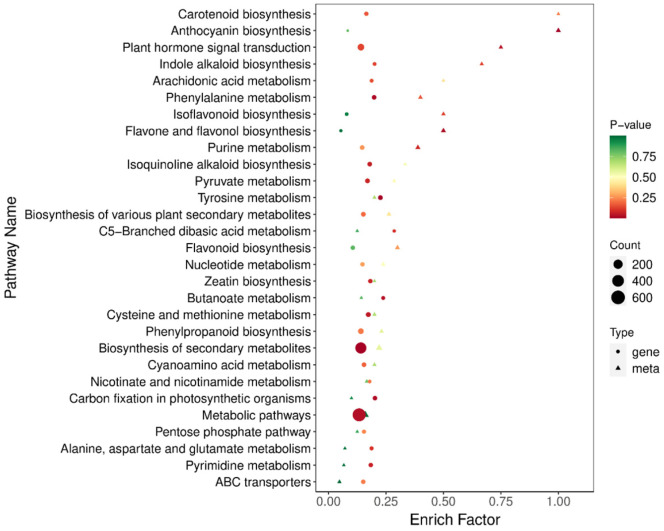
KEGG co-enrichment pathways of differentially accumulated metabolites (DAMs) and differentially expressed genes (DEGs) in the female flower tissues between ‘Chinese Ziguo’ (ZG) and ‘Silu Hongyu’ (HY).

**Figure 7:**
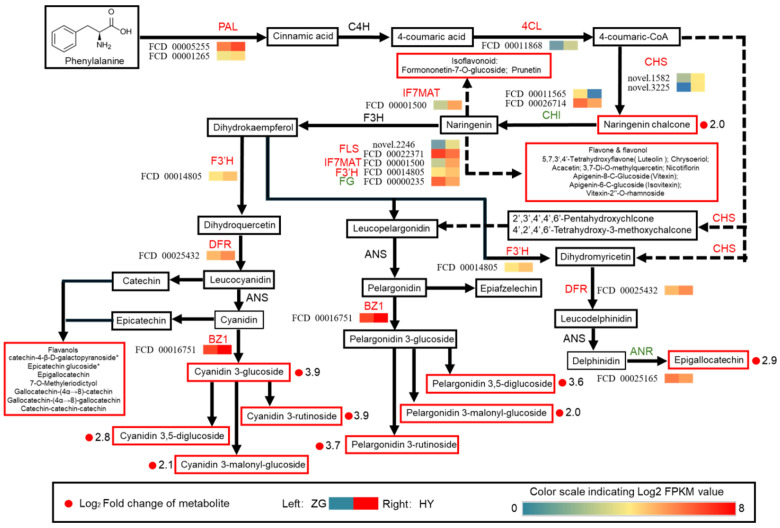
Transcript profiling of the anthocyanin and flavonoid pathway genes in the female flower tissues of two fig cultivars. The red solid circles represent log_2_fold change of metabolite (ZG vs. HY). Grids with colors from blue to red represent log_2_FPKM (ZG vs HY). ZG, ‘Chinese Ziguo’, HY, ‘Silu Hongyu’. Metabolites marked with asterisks are isomers.

To further decipher the mechanism underlying differential anthocyanin accumulation in the female flower tissues of ZG and HY, we performed KEGG co-enrichment analysis of DEGs and DAMs (Figure 6, Table S10). The anthocyanin biosynthesis pathway (ko00942) was co-enriched with eight DAMs and one DEG mapped to this pathway. Other flavonoid biosynthesis-related pathways, including phenylpropanoid biosynthesis (ko00940), flavonoid biosynthesis (ko00941), isoflavonoid biosynthesis (ko00943), flavone and flavonol biosynthesis (ko00944), were also co-enriched. Based on the changes in the content of DAMs and DEGs detected in this study, combined with these pathways, the proposed anthocyanin biosynthesis pathway in the female flower tissues of figs was reconstructed (Figure 7). As shown in Figure 7, several key structural genes involved in anthocyanin biosynthesis, such as *FcPAL*, *Fc4CL*, *FcCHS*, and *FcF3’H*, were significantly upregulated in the female flower tissues of HY compared with ZG. Consistently, the contents of multiple metabolites responsible for anthocyanin accumulation, such as naringenin chalcones, cyanidin 3-glucoside, and pelargonidin 3,5-diglucoside, were significantly increased in the HY female flower tissues (Figure 7). Overall, our results indicate that the differential expression of these structural genes involved in flavonoid biosynthesis, combined with the differential accumulation of metabolites in this pathway, collectively determines the different anthocyanin accumulation patterns in the female flower tissues of HY and ZG.

## 3. Discussion

Anthocyanins are key flavonoid pigments determining fruit color and nutritional value. Their biosynthesis pathway has been extensively studied in model plants and economically relevant crops, such as apple [10], cowpea pod [11], black radish [26], and Chinese cabbage [19]. Previous studies have reported the regulatory networks of fig peels and female flower tissues at different fruit development stages, as well as under ABA, light, and bagging treatments [4,5,27,28]. However, the complete picture of anthocyanin biosynthesis in figs, especially in the newly bred cultivars, remains poorly understood.

In this study, we found that HY exhibited higher anthocyanin content in the female flower tissues and possessed larger fruit size than ZG (Figure 1). A total of 350 DAMs were identified, among which 108 were flavonoids, including three chalcones, eight flavanones, 11 anthocyanins, 36 flavones, 23 flavonols, seven flavanols, 14 isoflavones and six other flavonoid compounds (Figure 3; Table S3). The anthocyanin biosynthesis (ko00942), flavone and flavonol biosynthesis (ko00944), isoflavonoid biosynthesis (ko00943), and flavonoid biosynthesis (ko00941) were significantly enriched (*p* < 0.05) (Figure 3D, Table S4). Notably, cyanidin-3-O-rutinoside, cyanidin-3-O-glucoside, peonidin-3,5-O-diglucoside, and cyanidin were the most abundant anthocyanins among the flavonoids [29]; the first two have been reported to be the major anthocyanins in ripened fig fruit [4,27,30,31]. All four metabolites exhibited higher accumulation levels in HY female flower tissues than in ZG (Table S3). Therefore, the increased accumulation of these metabolites contributes to the higher anthocyanin level in the female flower tissues of HY compared with ZG. It should be noted that the larger fruit size of HY may indirectly affect some metabolite concentrations. Nevertheless, the fact that these DAMs are restricted to flavonoid/anthocyanin pathways and are consistent with the expression patterns of key structural genes (Figure 4E and Figure 7; Table S6) argues against a simple, non-specific size effect.

Metabolomics alone only uncovers metabolic phenotypic differences in the female flower tissues between HY and ZG, leaving upstream regulatory events largely unknown. To clarify how anthocyanin composition and content correlate with the expression of structural genes and TFs in the biosynthesis pathway, we performed a transcriptomic analysis on the same tissues. The results showed that the phenylpropanoid biosynthesis (ko00940), anthocyanin biosynthesis (ko00942), flavonoid biosynthesis (ko00941), isoflavonoid biosynthesis (ko00943), and flavone and flavonol biosynthesis (ko00944) were identified (Figure 4D, Table S8) and were consistently enriched with DAMs at the metabolomic level (Figure 4D and Figure 6; Tables S8 and S10). Notably, isoflavonoids are prominently found in legumes but have also been isolated from numerous non-leguminous families, including Moraceae, Rosaceae, Asteraceae, Iridaceae, and Stemonaceae [32,33]. The enrichment of isoflavonoid biosynthesis pathway in figs (family Moraceae) is noteworthy, as it provides additional evidence for the presence and potential functionality of this pathway in a non-leguminous species. Nevertheless, the biological roles of isoflavonoids in Moraceae, especially in figs, remain rarely reported and warrant further investigation. Integrating transcriptomic and metabolomic data uncovered a key module positively linked to anthocyanin accumulation (Figure 7). Within this module, the expression levels of structural genes *FcPAL*, *Fc4CL*, *FcCHS*, *FcF3’H*, *FcIF7MAT*, *FcDFR*, and *FcBZ1* were significantly increased in the female flower tissues of HY compared with ZG (Figure 4E, Table S6). Previous studies have also demonstrated that they play an important role in the anthocyanin biosynthesis in the peel and female flower tissues of figs [4,5]. Notably, *BZ1* encodes a UDP-glucose flavonoid glucosyl-transferase (UFGT) that catalyzes anthocyanidin glucosylation to generate stable intermediates essential for pigment formation [34,35]. In the female flower tissues of HY, the upregulated expression of *BZ1* was accompanied by increased accumulation of its catalytic products, such as cyanidin 3-glucoside, cyanidin 3-rutinoside, pelargonidin 3.5-diglucoside, and pelargonidin 3-rutinosode (Figure 7; Table S9). Collectively, the differential expression of these structural genes and the altered accumulation of metabolites jointly shape the distinct patterns of anthocyanin accumulation in the female flower tissues of HY and ZG. The actual regulatory roles of these candidate genes warrant further functional validation in future studies.

Anthocyanin biosynthesis is a sophisticated and coordinated metabolic process that relies on the combined action of structural genes and regulatory TFs. Among these regulators, MYB, bHLH and WD40 family members have been well documented as core modulators of anthocyanin biosynthesis in a wide range of plant species [10,35,36]. For instance, AtMYBL2 negatively regulates the biosynthesis of anthocyanin by repressing the expression of *AtDFR* and *AtTRANSPARENTTESTA8* (*TT8*) genes in *Arabidopsis thaliana* [37]. FaMYB5 positively regulated the accumulation of anthocyanins through the trans-activation of the *FaF3’H* and *FaLEUCOANTHOCYANTIN REDUCTASE* (*LAR*) in strawberry [15]. MdbHLH3 promotes anthocyanin accumulation by interacting with MdMYB1 to directly activate the expression of *MdUFGT* in apple [38]. PybHLH3 interacts with PyWRKY26 to synergistically induce the transcript of *PyMYB114*, which further drives anthocyanin accumulation in pear [39]. In the present study, we found that 16 FcMYB and FcMYB-related TFs and 11 FcbHLH TFs exhibited differential expression in the female flower tissues between HY and ZG (Figure 4E; Table S6). Additionally, eight FcWRKY TFs were also differentially identified in the same tissues of the two fig cultivars (Figure 4E; Table S6). Previous studies have shown that AtWRKY33 negatively regulates anothcyanin accumulation by directly repressing the *AtDFR* expression in *A. thaliana* [40]; PbWRKY75 promoted pear anthocyanin biosynthesis by directly activating the expression of *PbDFR* and *PbUFGT* in pear [41]. Moreover, FcNAC, FcAP2, and FcbZIP TFs were also identified (Table S6). Hence, we speculated that the differential expression of these TFs may account for the distinct anthocyanin accumulation patterns in the female flower tissues between HY and ZG. Further studies are required to uncover the precise functions and molecular regulatory mechanisms of these TFs during anthocyanin accumulation in fig female flower tissues. In addition, time-series transcriptomic and metabolomic profiling across multiple developmental stages would help fully elucidate the temporal regulatory network underlying coloration in fig female flower tissues.

In conclusion, this study integrated transcriptomic and metabolomic analyses to explore the regulatory framework underlying female flower tissues coloration in two fig cultivars, HY and ZG. Differential accumulation of flavonoid metabolites, particularly anthocyanins, was the primary cause of the color divergence in the female flower tissues. Furthermore, distinct expression patterns were characterized for structural genes and TFs involved in the flavonoid biosynthesis pathway. Genetic modulation of these key structural genes and TFs provides a promising strategy to enrich flavonoid and anthocyanin contents in fig female flower tissues. This can further improve the nutritional quality and economic value of fig fruits.

## 4. Materials and Methods

### 4.1. Plant Materials

The fig cultivar ‘Chinese Ziguo’ (ZG) and ‘Silu Hongyu’ (HY) were grown in a commercial orchard of Yangling (34°15′23.8475” N, 108°1′54.5580” E), Shaanxi Province in China. Both cultivars were 3 years old and cultivated under identical environmental and agronomic conditions, including natural sunlight exposure, ambient temperature, uniform irrigation, fertilization, and pest management regimes, throughout the entire growth period in the same orchard. Female flower tissues used for the metabolomic profiling and RNA-seq were collected at about 75 days after fruit setting. For each cultivar, three biological replicates were designed. Each biological replicate was pooled from 20 fruits randomly harvested from 10 independent fig trees. The fruits were immediately transported to the laboratory, where the female flower tissues (approximately 2 mm thick) were carefully excised using a razor blade. The collected tissues were snap-frozen in liquid nitrogen, ground into a fine powder, and stored at −80 °C until further analysis.

### 4.2. Anthocyanin Determination

The total anthocyanin content was determined using a plant anthocyanin kit purchased from Suzhou Michy Biomedical Technology Co., Ltd. (Suzhou, China) via spectrophotometric method. The detection wavelength, standard calibration method and calculation formula were strictly followed according to the manufacturer’s instructions—anthocyanin content (µg/g) = 33.4 × ΔA × F ÷ FW, where ΔA = (A530 − A700)_Sample_ − (A530 − A700)_Control_, while F represents the dilution ratio, and FW is the fresh weight of the sample.

### 4.3. Metabolite Extraction

The biological samples were placed in a freeze-dryer (Scientz-100F, Ningbo, China) for vacuum freeze-drying and then ground into powder with a grinder (MM 400, Retsch, Stadt Hahn, Germany) at 30 Hz for 1.5 min. Subsequently, 50 mg of the resulting dry powder was dissolved in 1.2 mL of −20 °C pre-cooled 70% methanol with internal standard. To ensure a thorough suspension, the mixture was vortexed for 30 s, with a 30-min interval, and this process was repeated six times. After centrifugation (rotation speed 12,000 rpm, 3 min), the resulting supernatant was filtered using a microporous member with a pore size of 0.22 μm.

### 4.4. LC-MS/MS Acquisition Conditions

The data acquisition system comprised an ExionLC™ AD ultra-performance liquid chromatography (UPLC) system (https://sciex.com.cn/) and an Applied Biosystems 6500 QTRAP (SCIEX, Woodlands, Singapore) tandem mass spectrometry (MS/MS) system (https://sciex.com.cn/). The UPLC analysis was performed on an Agilent SB-C18 (1.8 µm, 2.1 mm × 100 mm) column. The mobile phase consisted of solvent A (pure water with 0.1% formic acid) and solvent B (acetonitrile with 0.1% formic acid). The injection volume was 2 μL. The sample measurements were performed with a gradient program that employed the starting conditions of 95% solvent A and 5% solvent B. Within 9 min, a linear gradient to 5% solvent A, 95% solvent B was programmed, and a composition of 5% solvent A, 95% solvent B was kept for 1 min. Subsequently, a composition of 95% solvent A and 5.0% solvent B was adjusted within 1.1 min and kept for 2.9 min. The flow velocity was set as 0.35 mL per minute; the column oven was set to 40 °C. The effluent was alternatively connected to an ESI-triple quadrupole-linear ion trap (QTRAP)-MS. The operation parameters of the ESI source were set as follows: source temperature at 500 °C; ion spray voltage (IS) at 5500 V in positive ion mode and −4500 V in negative ion mode; ion source gas I (GSI), gas II (GSII), and curtain gas (CUR) were adjusted to 50 psi, 60 psi, and 25 psi, respectively; collision-activated dissociation (CAD) was set to high. Triple quadrupole (QQQ) scans were performed as multiple reaction monitoring (MRM) experiments with collision gas (nitrogen) set to medium. The declustering potential (DP) and collision energy (CE) for each MRM transition were further optimized. A specific set of MRM transitions was monitored for each time period based on the metabolites eluted within that period.

### 4.5. Metabolite Identification and Quantification

Metabolite identification was based on the MetWare Database (MWDB) by matching accurate mass (MS, 20 ppm), MS^2^ fragments (20 ppm), isotopic distribution, and retention time (0.2 min). No authentic standards were used for absolute quantification of all identified metabolites. Relative quantification was performed using MRM.

### 4.6. Multivariate Statistical Analysis

Both HCA and PCA were carried out by R package complex heatmap. For HCA, normalized signal intensities of metabolites (unit variance scaling) are visualized as a color spectrum. Unsupervised PCA was performed by statistics function ‘prcomp’ in R v3.10.1 (www.r-project.org). The DAMs were determined by |log_2_FC|) > 1, VIP > 1, and *p* < 0.05. The data was log transform (log_2_) and mean centering before OPLS-DA. In order to avoid overfitting, a permutation test (200 permutations) was performed.

### 4.7. KEGG Annotation and Enrichment Analysis

Identified metabolites were annotated using KEGG Compound database (http://www.kegg.jp/kegg/compound/, accessed on 20 July 2022), annotated metabolites were then mapped to the KEGG pathway database (http://www.kegg.jp/kegg/pathway.html, accessed on 20 July 2022). Pathways with significantly regulated metabolites mapped to were then fed into MSEA (metabolite sets enrichment analysis); their significance was determined by the hypergeometric test’s *p*-values.

### 4.8. cDNA Library Construction and Sequencing

Total RNA was extracted using the RNeasy Plant Mini Kit (QIAGEN, Hilden, Germany). RNA degradation and contamination were monitored on 1% agarose gels. A NanoPhotometer spectrophotometer (IMPLEN, Munich, Germany) was used to check RNA purity. The RNA Nano 6000 Assay Kit of the Bioanalyzer 2100 system (Agilent, Santa Clara, CA, USA) was used to assess the integrity of RNA. RNA concentration was measured using Qubit^®^ RNA Assay Kit in Qubit^®^2.0 Flurometer (Thermo Fisher Scientific, Waltham, MA, USA). Sequencing libraries were generated using NEBNext^®^UltraTM RNA Library Prep Kit for Illumina^®^ (NEB, Ipswich, MA, USA) following the manufacturer’s recommendations, and index codes were added to attribute sequences to each sample. The cDNA libraries were sequenced on the Illumina sequencing platform by Metware Biotechnology Co., Ltd. (Wuhan, China). Clean reads were obtained by using fastp [42] to remove the reads containing adapter and poly-N sequences and low-quality reads from raw data. All subsequent analyses were based on clean data with high quality. Three biological replicates were performed for each sample.

### 4.9. Differential Gene Expression Analysis

Clean reads were mapped to the “Ficus_carica.UNIPI_FiCari_1.0.dna.toplevel.fa” reference genome using HISAT2 [43] with default parameters (https://plants.ensembl.org/Ficus_carica/Info/Index?db=core, accessed on 3 August 2022). Reads were assembled into transcripts using StringTie v1.3.4d. Gene alignment and FPKM were calculated using HISAT2 v2.1.0 and featureCounts v1.6, respectivitely1. DESeq2 v1.22.1 was used to analyze the differential expression between the two groups, and the *P* value was corrected using the Benjamini and Hochberg method [44,45]. Genes with |log_2_FC| ≥ 1 and *FDR* < 0.05 were defined as DEGs. GO and KEGG pathway enrichment analyses were performed using clusterProfiler v4.6.0, and the results were visualized via the Bioinformatics online platform (http://www.bioinformatics.com.cn/). The novel genes were subjected to sequence alignment against the KEGG, GO, NR, Swiss-Prot, TrEMBL, and KOG databases using DIAMOND v0.9.24.125 [46] to obtain annotation results, with the alignment parameter set to an E-value of 1 × 10^−5^ (Table S6). Transcription factors were predicted using iTAK software v1.7a [47].

### 4.10. RT-qPCR

Total RNA was isolated from female flower tissues at about 75 days after fruit setting using the SteadyPure Plant RNA Extraction Kit (Accurate Biology, Changsha, China) and were subsequently reverse-transcribed into cDNA (TransGen, Beijing, China). RT-qPCR was performed on the QuantStudioTM 7 Flex Real-Time System (Thermo Fisher Scientific, Waltham, MA, USA) with SYBR Green Master Mix (Cofitt, Hongkong, China). Three biological replications were performed, each with three technical replications. The fig *Actin* gene was used as the internal reference, and relative expression levels of target genes were normalized to *Actin* and calculated using a modified 2^−ΔΔCt^ method. Primer sequences are listed in Table S11.

## Data Availability

The data presented in this study are available in the article and the Supplementary Materials. The raw RNA-seq data have been deposited in the Genome Sequence Archive (GSA) at the China National Center for Bioinformation (CNCB) under accession number PRJCA065562. The metabolomics data are available from the corresponding author upon reasonable request.

## Supplementary Materials

The following supporting information can be downloaded at: https://www.mdpi.com/article/10.3390/genes17060694/s1: Table S1. Identification and classification of metabolites in the female flower tissues of HY and ZG; Table S2. Differentially accumulated metabolites (DAMs) identified in the female flower tissues of HY and ZG (|log_2_FC| ≥ 1, VIP > 1); Table S3. Differentially accumulated flavonoids identified in the female flower tissues of HY and ZG (|log_2_FC| ≥ 1 and VIP > 1); Table S4. KEGG enrichment analysis of differentially accumulated metabolites in the female flower tissues between ZG and HY; Table S5. RNA-seq data statistic; Table S6. A list of differentially expressed genes (DEGs) in female flower tissues between two fig varieties ZG and HY (log_2_|FC| ≥ 1 and *FDR* < 0.05); Table S7. Differentially expressed genes (DEGs) involved in flavonoid biosynthesis in the female flower tissues between ZG and HY; Table S8. KEGG enrichment analysis of differentially expressed genes (DEGs) in the female flower tissues between ZG and HY; Table S9. Correlation analysis between transcripts and metabolites; Table S10. KEGG co-enrichment analysis of differentially expressed genes (DEGs) and differentially accumulated metabolites (DMAs) in the female flower tissues between ZG and HY; Table S11. List of primers used for RT-qPCR expression analysis.
